# Supplementary material for: Tumor detection by analysis of both symmetric- and hemi-methylation of plasma cell-free DNA
Source: Nat Commun. 2024 Jul 20;15:6113. doi: 10.1038/s41467-024-50471-1 (PMC11271492; doi:10.1038/s41467-024-50471-1)
Supplement: Supplementary file 1 — Supplementary Information [file 41467_2024_50471_MOESM1_ESM.pdf]

Tumor detection by analysis of both symmetric- and hemi-methylation of plasma cell-free DNA

Xu Hua[1,2,3,4#], Hui Zhou[1,2,3,4#], Hui-Chen Wu,[2,5], Julia Furnari[2,6], Corina P Kotidis[2,7], Raul Rabadan[8], Jeanine M. Genkinger[2,9], Jeffrey N. Bruce[2,6], Peter Canoll[2,7], Regina M. Santella[2,5], and Zhiguo Zhang[1,2,3,4,*]

[1]Institute for Cancer Genetics, Columbia University Irving Medical Center, New York, NY 10032, USA

[2]Herbert Irving Comprehensive Cancer Center, Columbia University Irving Medical Center, New York, NY 10032, USA

[3]Department of Pediatrics, Columbia University Medical Center, New York, NY 10032, USA

[4]Department of Genetics and Development, Columbia University Medical Center, New York, NY 10032, USA.

[5]Department of Environmental Health Sciences, Mailman School of Public Health, Columbia University, New York, NY 10032, USA

[6]Department of Neurological Surgery, Columbia University Irving Medical Center, New York, NY 10032, USA

[7]Department of Pathology and Cell Biology, Columbia University Irving Medical Center, New York, NY 10032, USA

[8] Program for Mathematical Genomics and Department of Systems Biology, Columbia University Irving Medical Center, New York, NY, USA

[9]Department of Epidemiology, Columbia University Mailman School of Public Health, New York, NY, USA

# These authors contribute equally to this work.

*Corresponding author: Zhiguo Zhang, zz2401@cumc.columbia.edu

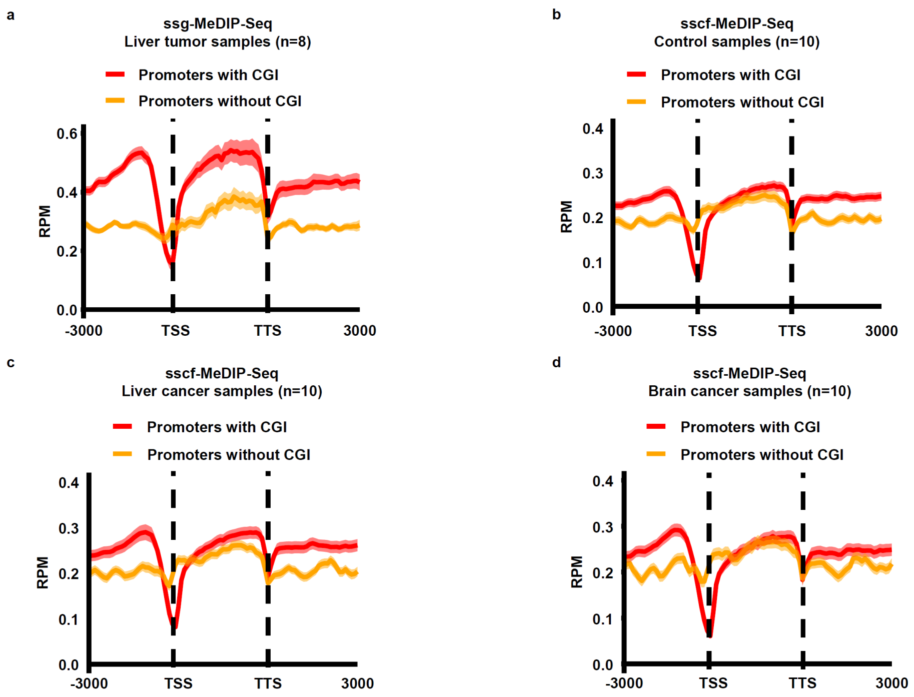

Figure S1. DNA methylation density surrounding genes measured by ssg-MeDIP-Seq and sscf-MeDIP-Seq.

(**a**) The average genomic DNA methylation density surrounding genes (3000bp upstream of transcription start site (TSS) and 3000bp downstream transcription termination site (TTS) in eight liver tumor samples measured by ssg-MeDIP-Seq.
(**b-d**) The average cfDNA methylation density surrounding genes in 10 control samples (b), 10 liver cancer samples (c) and 10 brain tumor samples based on sscf-MeDIP-Seq analysis. Genes are grouped those with (13,553) and without (6,713) CpG islands (CGI) at their promoters. TSS: transcription start site, TTS: transcription termination site. Data are represented as mean±95% confidence interval.

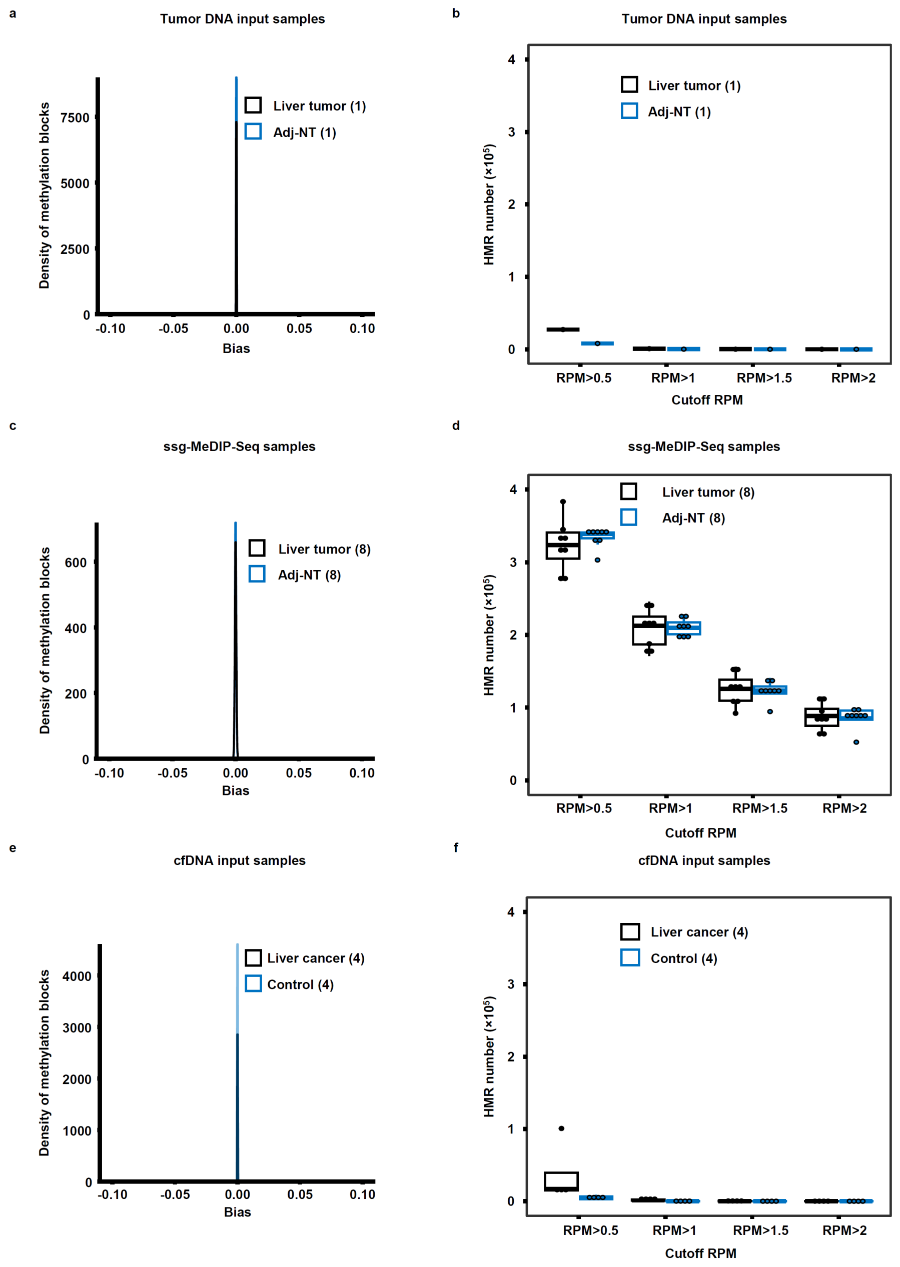

Figure S2: Analysis of strand bias at ~2 M methylation block of both input and ssg-MeDIP-Seq samples. (**a**) The distribution of ~2M methylation blocks with different strand bias of two genomic DNA input samples. The bias was calculated using the formula (W-C)/(W+C) as in Fig. 2a. (**b**) Effects of different RPM cutoffs on the number of blocks showing bias>0.3 of two input samples of genomic DNA from liver tumor and Adj-NT. (**c**) The distribution of ~2M methylation blocks with different bias of eight ssg-MeDIP-Seq datasets of liver tumor DNA and corresponding Adj-NT. (**d**). Effects of different RPM cutoffs on the number of HMRs of eight liver tumor DNA and eight Adj-NT ssg-MeDIP-Seq samples. (**e**) The distribution of ~2M

methylation blocks with different bias of eight cfDNA input samples from four liver tumor and four controls. (**f**) Effects of different RPM cutoffs on the number of blocks showing HM (bias>0.3) of four liver cancer cfDNA and four control cfDNA samples. Box plots in b, d and f show the median, 25% and 75% quartiles, minimal and maximal values of HMR number.

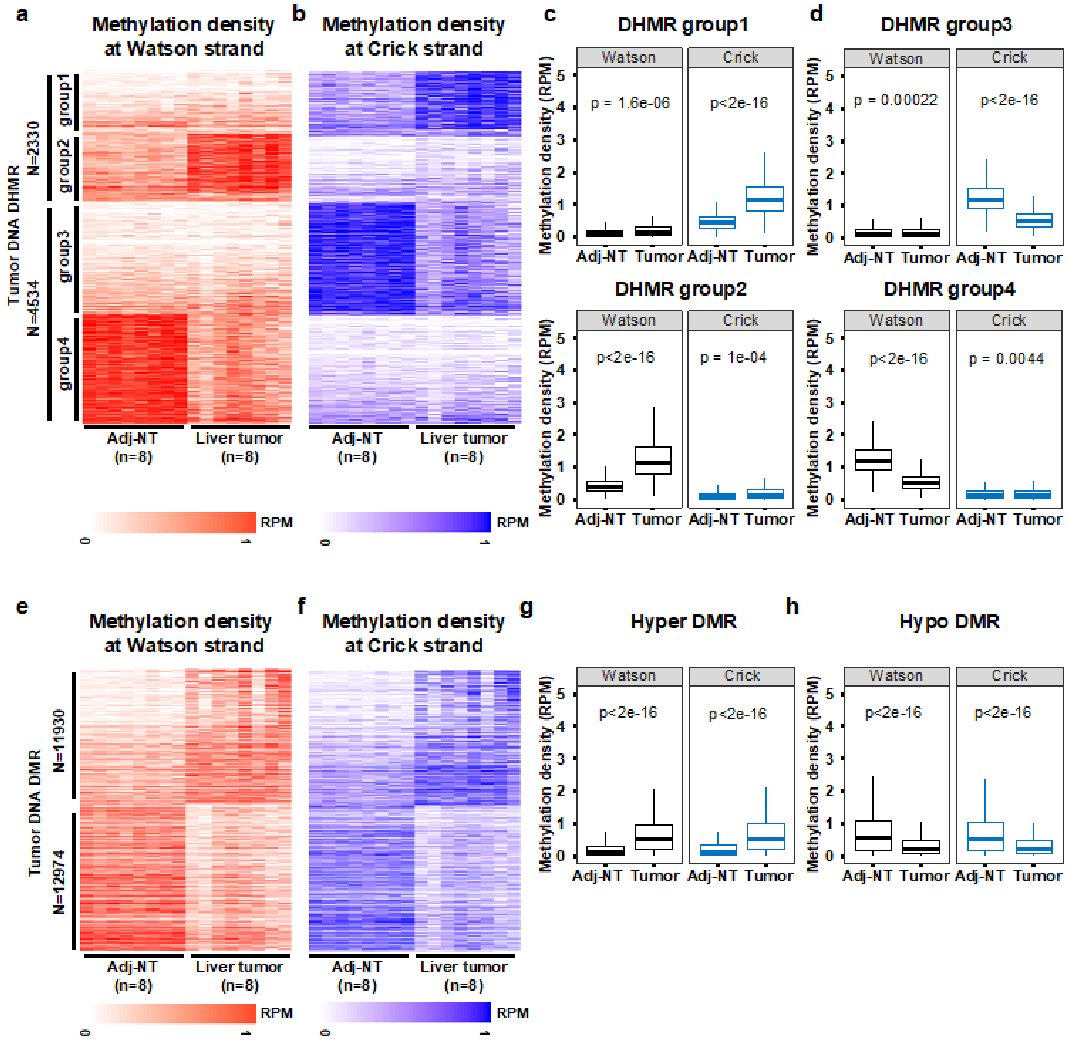

Figure S3: DNA methylation density of Watson or Crick strand at DHMRs and DMRs of 8 liver tumor DNA samples compared to their corresponding Adj-NT controls.

(**a-b**) Heatmaps of DNA methylation density of Watson (a) and Crick strand (b) at 6,864 liver tumor DHMRs compared to Adj-NT controls identified in Figure 2. These DHMRs could be separated into 4 groups based on increased hemi-methylation (HM) of Watson strand (group 2) or Crick strand (group 1) or reduced HM of Watson (group 4) or Crick strand (group 3).

(c-d) The average DNA methylation density at Watson or Crick strand at each of the 4 groups of DHMRs. Box plots show the median, 25% and 75% quartiles, minimal and maximal values of methylation density with p values calculated by T-test. 8 Adj-NT and 8 tumor sample were compared in the box plots.

(**e-f)** Heatmaps of DNA methylation density of Watson (e) and Crick strand (f) at 24,904 liver tumor DMRs compared to Adj-NT controls identified in Figure 1.

(**g-h**) The average DNA methylation density of Watson and Crick strand at hyper-methylated DMRs and hypo-methylated DMRs in liver tumor samples compared to controls. Box plots show the median, 25% and 75% quartiles, minimal and maximal values of methylation density with p values by T-test. 8 Adj-NT and 8 tumor sample were compared in the box plots.

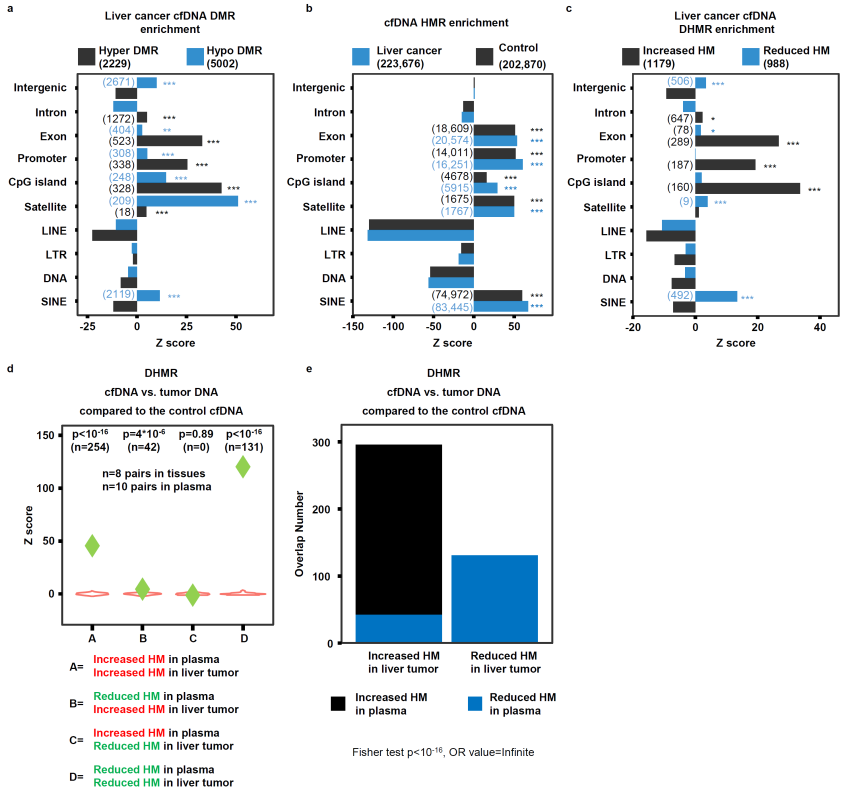

Figure S4. Properties of liver tumor DNA and liver tumor plasma cfDNA DMRs and DHMRs.

(**a-c**) The enrichment of liver cancer cfDNA DMR (a), cfDNA HMR (b) and liver cancer cfDNA DHMR (c) compared to controls. (a) Hyper- and hypo-differentially methylated regions of liver cancer cfDNA are annotated separately. (b) Hemi-methylated regions (HMR) of liver cancer cfDNA and control cfDNA samples are annotated separately. (c) Differentially hemi-methylated regions (DHMR) of liver cancer cfDNA samples compared to control samples with increased and reduced hemi-methylation are annotated separately. The interested region is firstly overlapped with each annotated locus, then compared with the overlapped number in random distribution and calculated the Z score. The p value was computed by the random distribution in a one-sided way and no multiple comparison correction was performed. The significantly enriched locus is labelled with asterisks shown with corresponding color, with the number of overlapped DMRs or DHMRs shown in parenthesis. "*" indicates $p<0.05$; "**" indicates $p<0.01$; "***" indicates $p<0.001$.

(**d-e**) Violin plots (d) and bar graph (e) showing the overlaps between liver cancer cfDNA DHMRs and liver cancer DNA DHMRs. Liver cancer cfDNA DHMRs and tumor DNA DHMRs were identified using the same control cfDNA samples. Violin plots represent the random distribution of overlaps from 100 permutations and p values were computed by the random

permutation distribution in a one-sided way, with green diamonds being observed overlaps. The statistical analysis for the bar plot was performed using the Fisher test in a two-sided way.

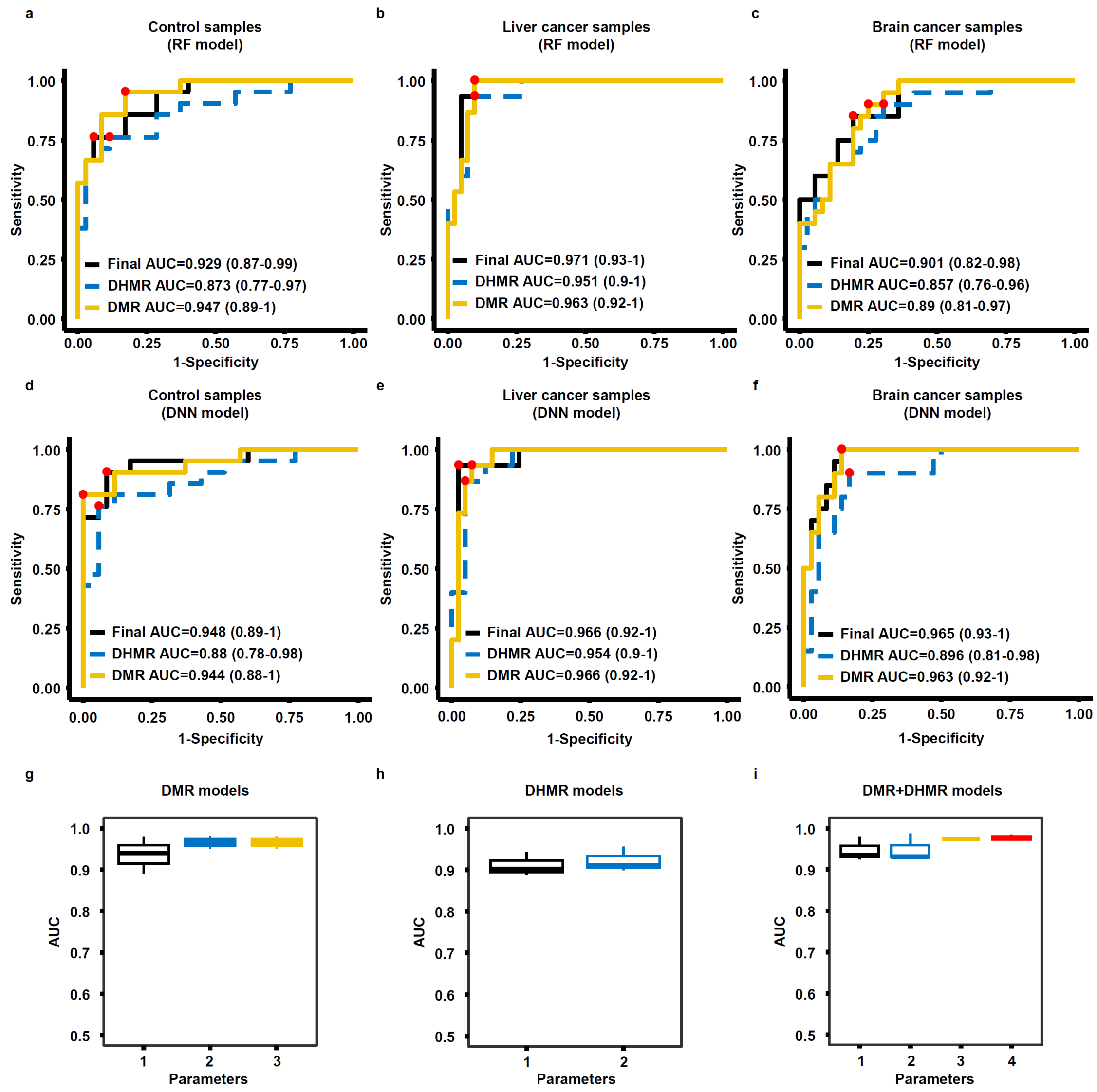

Figure S5. Evaluation of prediction performance using different machine learning algorisms and parameters for tuning.

(**a-c**) Evaluation of the performance of random forest models in validation cohort using the ROC curve on the control samples (a), liver cancer samples (b) and brain cancer samples (c) using cfDNA DMR, DHMR or DMR+DHMR as inputs for modeling training.

(**d-f**) Evaluating the performance of deep neural network models for prediction of control cfDNA samples (d), liver cancer cfDNA samples (e) and brain cancer cfDNA samples (f) in the validation cohort. Models were trained using DMRs, DHMRs or DMRs+DHMRs.

(**g**) AUROC values of the validation cohort samples predicted using GLMNET models trained with DMRs using different cutoff parameters: p=0.05, LFC=0.58; p=0.05, LFC=1; p=0.01, LFC=1. LFC: log fold changes in DNA methylation density based on sscf-MeDIP-Seq signals.
(**h**) AUROC values of different GLMNET models trained using DHMRs selected from different cutoffs: p=0.05, feature importance=0; p=0.01, feature importance=0.
(**i)** AUROC values of the validation cohort samples predicted using GLMNET models trained with DMR and DHMRs with different cutoffs: parameters 1: DMR p=0.05, LFC=0.58, DHMR p=0.05; parameters2: DMR p=0.05, LFC=0.58, DHMR p=0.01; parameters3: DMR p=0.01, LFC=1, DHMR p=0.05; parameters4: DMR p=0.01, LFC=1, DHMR p=0.01.
Box plots show the median, 25% and 75% quartiles, minimal and maximal values of AUC values and each box shows the AUC values of 30 models for 3 sample groups.

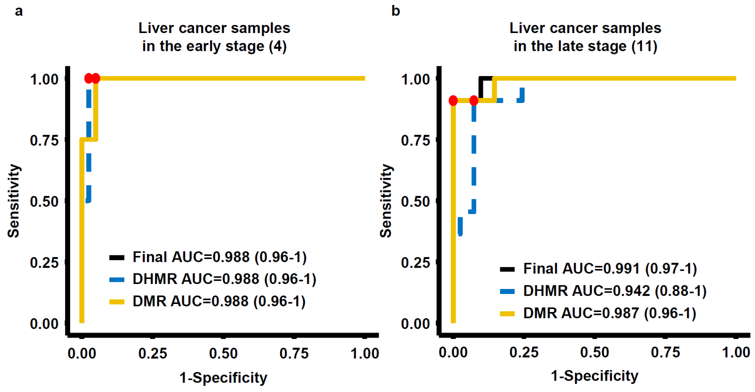

Figure S6. Prediction of early (a) and late stage (b) of liver cancer samples in the training cohort using DMR-, DHMR-, DMR+DHMR-based models. ROC curves of 4 early stage and 11 late stage samples were shown. The 95% confidence interval of AUC for each model is labeled in parenthesis.

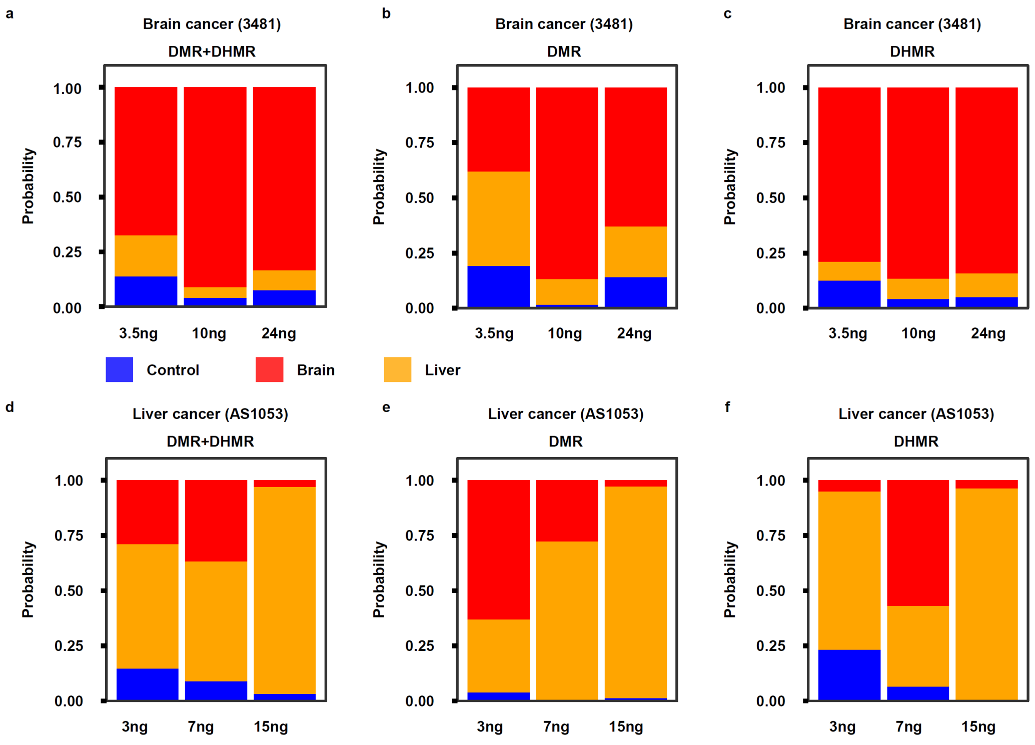

Figure S7. Evaluate the amount of cfDNA needed to generate high quality of sscf-MeDIP-Seq datasets.

(**a-c**) Prediction probability using sscf-MeDIP-Seq datasets generated from three different amount of one brain tumor cfDNA sample using the models trained with DMRs+DHMRs (a), DMRs (b) and DHMRs (c).
(**d-f**) Prediction probability using sscf-MeDIP-Seq datasets generated from three different amount of one liver tumor cfDNA sample using the models trained with DMRs+DHMRs (e), DMRs (f) and DHMRs (g). Red, yellow and blue represent the probability of brain cancer, liver cancer or healthy normal, respectively.

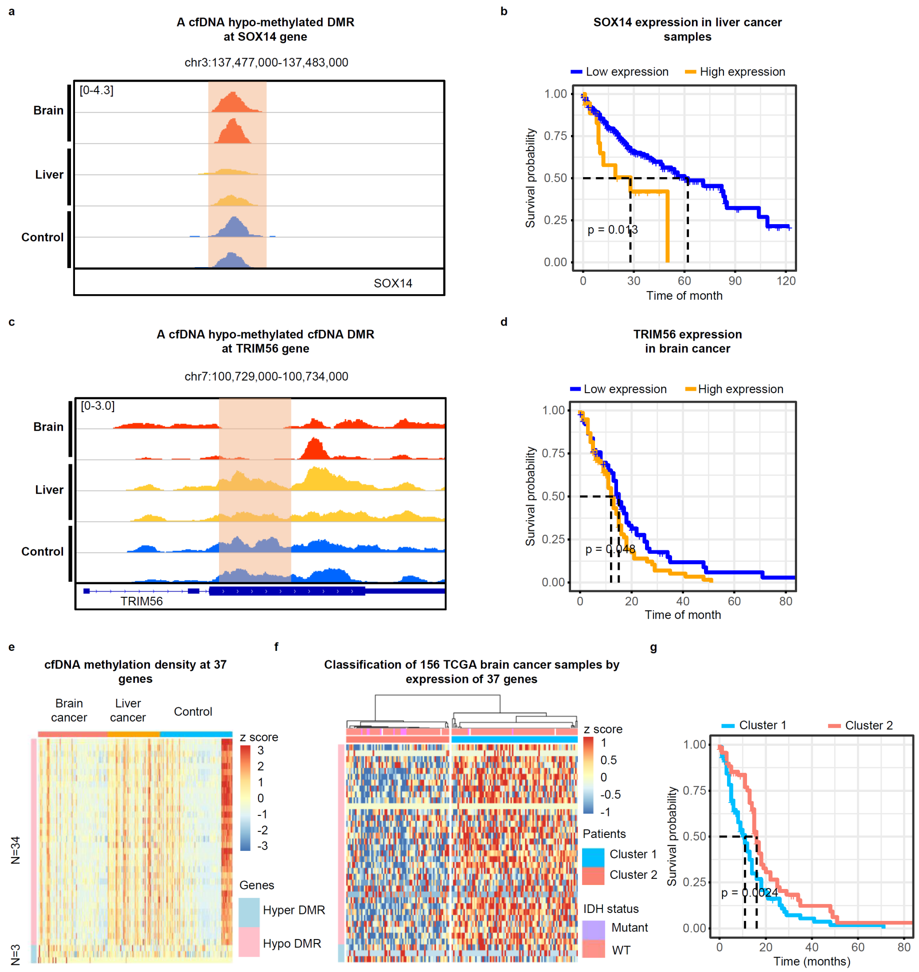

Figure S8. Potential relationship between cfDNA DMRs and the expression of their closest genes in tumor tissue samples.

(**a**) A snapshot of hypomethylated DMR surrounding SOX14 gene in liver cancer compared to control and brain tumor samples.

(**b**) Survival analysis of 371 liver cancer samples in the TCGA database separated into two groups based on the median expression of SOX14 in these 371 samples.

(**c**) A snapshot of brain cancer cfDNA hypomethylated DMR at the TRIM56 gene locus.

(**d**) Survival analysis of 156 patients separated into two groups based on the median expression of TRIM56.

(**e**) cfDNA methylation density at the 37 genes with at least one brain cancer cfDNA DMR nearby and with their expression in brain cancer tissue being associated with patient survival. The z-score, represented by color, is log2 (RPKM) of sscf-MeDIP-Seq signals. A "Hyper DMR" refers to a gene with at least one hyper-methylated cfDNA DMR nearby. A "Hypo DMR" is defined as a gene with a hypo-methylated cfDNA DMR nearby.

(**f**) Classification of 156 brain tumor samples in the TCGA-GBM cohort based on expression of the 37 genes identified above. The color represents the z-score of log2 (RPKM) of 37 genes in brain cancer samples based on RNA-seq.

(**g**) Kaplan–Meier survival analysis of 156 brain cancer patients in two clusters defined by the expression of 37 genes. P value is calculated by log rank test.
