# Supplementary material for: Tumor detection by analysis of both symmetric- and hemi-methylation of plasma cell-free DNA
Source: Nat Commun. 2024 Jul 20;15:6113. doi: 10.1038/s41467-024-50471-1 (PMC11271492; doi:10.1038/s41467-024-50471-1)
Supplement: Supplementary file 3 — Reporting Summary [file 41467_2024_50471_MOESM3_ESM.pdf]

nature portfolio

Corresponding author(s): Zhiguo Zhang

Last updated by author(s): Jan 24, 2024

Reporting Summary

Nature Portfolio wishes to improve the reproducibility of the work that we publish. This form provides structure for consistency and transparency in reporting. For further information on Nature Portfolio policies, see our Editorial Policies and the Editorial Policy Checklist.

Statistics

For all statistical analyses, confirm that the following items are present in the figure legend, table legend, main text, or Methods section.

n/a	Confirmed	
☐	☒	The exact sample size (*n*) for each experimental group/condition, given as a discrete number and unit of measurement
☐	☒	A statement on whether measurements were taken from distinct samples or whether the same sample was measured repeatedly
☐	☒	The statistical test(s) used AND whether they are one- or two-sided<br>*Only common tests should be described solely by name; describe more complex techniques in the Methods section.*
☐	☒	A description of all covariates tested
☐	☒	A description of any assumptions or corrections, such as tests of normality and adjustment for multiple comparisons
☐	☒	A full description of the statistical parameters including central tendency (e.g. means) or other basic estimates (e.g. regression coefficient) AND variation (e.g. standard deviation) or associated estimates of uncertainty (e.g. confidence intervals)
☐	☒	For null hypothesis testing, the test statistic (e.g. *F*, *t*, *r*) with confidence intervals, effect sizes, degrees of freedom and *P* value noted<br>*Give P values as exact values whenever suitable.*
☐	☒	For Bayesian analysis, information on the choice of priors and Markov chain Monte Carlo settings
☐	☒	For hierarchical and complex designs, identification of the appropriate level for tests and full reporting of outcomes
☐	☒	Estimates of effect sizes (e.g. Cohen's *d*, Pearson's *r*), indicating how they were calculated

Our web collection on statistics for biologists contains articles on many of the points above.

Software and code

Policy information about availability of computer code

Data collection	MeDIP-Seq datasets were generated using procedures and samples from Columbia University Irving Medical Center (CUIMC).
Data analysis	R package "caret_6.0-86" was used to train the machine learning models. Custom code was uploaded to https://github.com/clouds-drift/plasma_MCD

For manuscripts utilizing custom algorithms or software that are central to the research but not yet described in published literature, software must be made available to editors and reviewers. We strongly encourage code deposition in a community repository (e.g. GitHub). See the Nature Portfolio guidelines for submitting code & software for further information.

Data

Policy information about availability of data

All manuscripts must include a data availability statement. This statement should provide the following information, where applicable:

- Accession codes, unique identifiers, or web links for publicly available datasets
- A description of any restrictions on data availability
- For clinical datasets or third party data, please ensure that the statement adheres to our policy

All data are uploaded to dbGaP database with ID of phs003462.v1.p1, entitled "Sensitive and accurate tumor detection by methylation and hemi-methylation of plasma cell-free DNA". Source data are provided with this paper.

Research involving human participants, their data, or biological material

Policy information about studies with human participants or human data. See also policy information about sex, gender (identity/presentation), and sexual orientation and race, ethnicity and racism.

Reporting on sex and gender	For cancer detection, samples from equal numbers of male and female participants were included for model training by a random selection.<br>Sex information was collected from Columbia University Irving Medical Center (CUIMC) obtained from medical records.<br>Written informed consent was obtained from all participants by the Columbia University Institutional Review Board.
Reporting on race, ethnicity, or other socially relevant groupings	Race, ethnicity or other socially relevant information were not included in this study.
Population characteristics	No other covariate-relevant population characteristics were used in this study.
Recruitment	Hepatocellular cancer patients' plasma samples were from an IRB-approved, hospital-based prospective study conducted at Columbia University Irving Medical Center (CUIMC) that recruited liver cancer patients (>18 years older) from Oct., 2008 to July 2014. Plasma samples from brain cancer patients were collected as part of an IRB-approved protocol to collect, bank and distribute de-identified samples from brain tumor patients at CUIMC. Subjects without cancer were recruited from advertisements around CUIMC also with IRB approval.
Ethics oversight	IRB of Columbia University Irving Medical Center (CUIMC)

Note that full information on the approval of the study protocol must also be provided in the manuscript.

Field-specific reporting

Please select the one below that is the best fit for your research. If you are not sure, read the appropriate sections before making your selection.

[x] Life sciences   [ ] Behavioural & social sciences   [ ] Ecological, evolutionary & environmental sciences

For a reference copy of the document with all sections, see nature.com/documents/nr-reporting-summary-flat.pdf

Life sciences study design

All studies must disclose on these points even when the disclosure is negative.

Sample size	271 samples were included in this study, and we analyzed as many samples as as we can obtain.
Data exclusions	No exclusion.
Replication	During initial test of methods, replications are good. for most samples analyzed, no repeats were performed because it is not necessary to repeat analysis of all 271 samples.
Randomization	We randomly choose 80% of 271 samples (215 samples) to identify DMR, DHMR and to train machine learning models.
Blinding	In the analysis, 56 samples in the validation cohort are blinding. Samples in the training cohorts used for machine learning are not.

Reporting for specific materials, systems and methods

We require information from authors about some types of materials, experimental systems and methods used in many studies. Here, indicate whether each material, system or method listed is relevant to your study. If you are not sure if a list item applies to your research, read the appropriate section before selecting a response.

Materials & experimental systems

n/a	Involved in the study	
[ ]	[x]	Antibodies
[x]	[ ]	Eukaryotic cell lines
[x]	[ ]	Palaeontology and archaeology
[x]	[ ]	Animals and other organisms
[ ]	[x]	Clinical data
[x]	[ ]	Dual use research of concern
[x]	[ ]	Plants

Methods

n/a	Involved in the study	
[x]	[ ]	ChIP-seq
[x]	[ ]	Flow cytometry
[x]	[ ]	MRI-based neuroimaging

Antibodies

Antibodies used	Antibodies against 5-mC (33D3) were purchased from Diagenode (C15200081). 0.6 ug anti-5-mC monoclonal antibody/200 ul reaction was used for MeDIP and then 1 ul of bridge antibody (Active Motif 53017) /200 ul.
Validation	After MeDIP-Seq, we compared the distribution of DNA methylation genome wide based on our datasets to that published ones to validate the procedures and antibodies used for MeDIP-Seq.

Clinical data

Policy information about clinical studies

All manuscripts should comply with the ICMJEguidelines for publication of clinical research and a completedCONSORT checklist must be included with all submissions.

Clinical trial registration	*Provide the trial registration number from ClinicalTrials.gov or an equivalent agency.*
Study protocol	*Note where the full trial protocol can be accessed OR if not available, explain why.*
Data collection	*Describe the settings and locales of data collection, noting the time periods of recruitment and data collection.*
Outcomes	*Describe how you pre-defined primary and secondary outcome measures and how you assessed these measures.*
